# The Formation of Methyl Ketones during Lipid Oxidation at Elevated Temperatures

**DOI:** 10.3390/molecules26041104

**Published:** 2021-02-19

**Authors:** Sandra Grebenteuch, Clemens Kanzler, Stefan Klaußnitzer, Lothar W. Kroh, Sascha Rohn

**Affiliations:** 1 Food Chemistry and Analysis, Technische Universität Berlin, Gustav-Meyer-Allee 25, 13355 Berlin, Germany; sandra.grebenteuch@tu-berlin.de (S.G.); clemens.kanzler@tu-berlin.de (C.K.); s.klaussnitzer@tu-berlin.de (S.K.); lothar.kroh@tu-berlin.de (L.W.K.); 2 Institute for Food and Environmental Research e. V., Papendorfer Weg 3, 14806 Bad Belzig, Germany; 3 NutriAct-Competence Cluster Nutrition Research, c/o The German Institute of Human Nutrition Potsdam-Rehbrücke, Arthur-Scheunert-Allee 114-116, 14558 Nuthetal, Germany

**Keywords:** methyl ketones, lipid oxidation, heptan-2-one, headspace GC-MS analysis, secondary and tertiary lipid oxidation products, phosphatidylethanolamine

## Abstract

Lipid oxidation and the resulting volatile organic compounds are the main reasons for a loss of food quality. In addition to typical compounds, such as alkanes, aldehydes and alcohols, methyl ketones like heptan-2-one, are repeatedly described as aroma-active substances in various foods. However, it is not yet clear from which precursors methyl ketones are formed and what influence amino compounds have on the formation mechanism. In this study, the formation of methyl ketones in selected food-relevant fats and oils, as well as in model systems with linoleic acid or pure secondary degradation products (alka-2,4-dienals, alken-2-als, hexanal, and 2-butyloct-2-enal), has been investigated. Elevated temperatures were chosen for simulating processing conditions such as baking, frying, or deep-frying. Up to seven methyl ketones in milk fat, vegetable oils, and selected model systems have been determined using static headspace gas chromatography-mass spectrometry (GC-MS). This study showed that methyl ketones are tertiary lipid oxidation products, as they are derived from secondary degradation products such as deca-2,4-dienal and oct-2-enal. The study further showed that the position of the double bond in the precursor compound determines the chain length of the methyl ketone and that amino compounds promote the formation of methyl ketones to a different degree. These compounds influence the profile of the products formed. As food naturally contains lipids as well as amino compounds, the proposed pathways are relevant for the formation of aroma-active methyl ketones in food.

## 1. Introduction

Lipid oxidation, along with the Maillard reaction, is one of the most important reactions in food chemistry. Whereas the Maillard reaction leads to the formation of aroma, flavor, and color, resulting from the reaction of sugar and amino compounds and being, in most cases, associated with positive attributes [1,2], lipid oxidation is associated with a loss of nutritional value of foods because the essential functions of omega-3 and omega-6 fatty acids are reduced [3,4]. These polyunsaturated fatty acids (PUFA) tend to be oxidized faster than saturated fatty acids by atmospheric oxygen and thermal influence due to their penta-1,4-diene structure [5].

In the course of lipid oxidation, hydroperoxides are formed as primary products due to oxidative, thermal, and hydrolytic reactions [6]. These peroxides are odorless and tasteless but decompose, as a result of further β-cleavage, into a variety of secondary degradation products of different compound classes such as alkanes, alcohols, esters, aldehydes, and ketones [7]. Some of these volatile compounds (VOC), for example hexanal, oct-2-enal and deca-2,4-dienal, are aroma-active, even in low concentrations [8]. They can cause significant sensorial changes leading to a reduced acceptability of certain foods [4,9,10]. In addition to unpleasant sensory qualities, some volatile aldehydes can also have a negative impact on human health. This has been especially shown for the unsaturated alk-2-enals and alka-2,4-dienals [11,12].

The formation mechanisms of most secondary VOC like hexanal are largely well understood [13]. However, the formation of alkan-2-ones (methyl ketones) is repeatedly described in various lipid-rich foods, but their origin or their precursors are unknown or are usually not discussed in detail. Their sensory attributes have been described as soapy, musty [14], paint, green, fruity [15], or ‘blue cheese’ [16]. They could be detected in mayonnaise [17], blue cheese [16], beans [18], roasted duck [14], baked potatoes [15], camembert cheese [19], dry cured ham [20], mitten crab [21], oat [22], sunflower oil [23], corn oil [24], soybean oil [25] and milk fat [26,27].

Some authors described that methyl ketones are formed from saturated fatty acids after their oxidation at high temperatures from an intermediary 3-oxo acids (formerly known as β-keto acids) by decarboxylation of the latter [28]. Especially in milk fat 3-oxo esters (β-keto esters), which are esterified to glycerol, have been identified as precursors for methyl ketones [29]. Furthermore, it has been shown that with increasing water content, the amount of methyl ketones also increased in milk fat [30,31].

However, the described pathway involving the decarboxylation of 3-oxo acids does not correspond to the methyl ketones found in milk fat. As an example, octanoic acid or octanoic acid esters in milk fat should result in the two C-atoms shortened hexan-2-one, because of β-oxidation. But in milk fat, almost exclusively methyl ketones with an odd number of carbon atoms, like heptan-2-one are formed, which does not correspond to the fatty acid composition of milk fat [32]. This inconsistency was also mentioned by Langler and Day (1964), but without discussing it in detail [27].

Besides milk fat, methyl ketone formation is poorly described in the literature. Gardner and Selke (1984) characterized the formation of heptan-2-one directly from the 13-hydroperoxide of linoleic acid via a 12,13-epoxide-11-oxyl radical [33]. Farmer et al. (1989) observed that heptan-2-one, octan-2-one, and decan-2-one were increasingly produced from the Maillard reaction of ribose with cysteine in the presence of phospholipids. However, as that study focused on the Maillard reaction and the evaluation of the flavor, the reason for the formation of methyl ketones was not described comprehensively [34].

The aim of the present study was to unravel the formation pathways of methyl ketones, their formation from different potential precursors such as linoleic acid, alka-2,4-dienals, alk-2-enals, and aldehydes in combination with different food-relevant amino compounds. The model systems focused, on the one hand, on typical lipid oxidation products and on the other hand on different structural characteristics such as alkyl chain length or position of double bonds. In addition to the model systems, selected fats and vegetable oils were heated at elevated temperatures to observe their potential in methyl ketone formation in food processes like baking, frying, or deep-frying.

## 2. Results and Discussion

### 2.1. Formation of Methyl Ketones in Selected Fats and Vegetable Oils

To get an overview of the formation of methyl ketones in foods, various typical fats and vegetable oils from local commercial suppliers were heated at 160 °C for 20 min. This temperature was chosen, as such elevated temperatures are reached during food processing, for example, baking, frying, or deep-frying even under household conditions. Using static headspace sampling, it was possible to detect methyl ketones in the homologous series from pentan-2-one to undecan-2-one, alongside of typical lipid oxidation products such as hexanal and various aldehydes (e.g., hept-2-enal, oct-2-enal, deca-2,4-dienal). The most common methyl ketones were hexan-2-one, heptan-2-one and octan-2-one, which could be detected in almost all samples except for butter, coconut fat and the liquid vegetable fat preparation II (Table 1). 

Obviously, the vegetable oils investigated differed in their fatty acid composition, but no correlation with the methyl ketones formed was observed within different ratios of saturated, monounsaturated and PUFA. As expected, in coconut fat, which mainly contains saturated fatty acids, no methyl ketones were detected. However, most methyl ketones were found in clarified butter, which contains hardly any water compared to butter. This is in contrast to the observations of other authors, who described that water removal leads to a reduced formation of methyl ketones [30,31]. Furthermore, higher concentrations of methyl ketones were detected in the liquid vegetable fat preparation I, containing whey ingredients compared to the liquid vegetable fat preparation II, which was without whey. This leads to the assumption that amino compounds could be involved in the formation of the methyl ketones. All in all, there was no consistent relationship between saturated fatty acids composition and the length of the alkyl chain of the methyl ketones formed.

**Figure 1:**
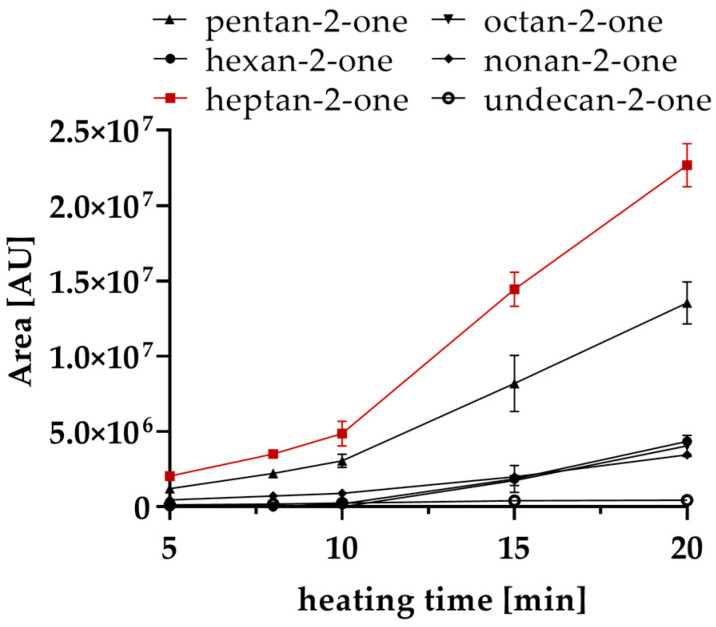
Peak area of six different methyl ketones in 1 g clarified butter heated at 160 °C for 20 min and analyzed by headspace gas chromatography mass spectrometry (HS-GC-MS).

Due to this fact and the fact that there was not any formation of methyl ketones in coconut oil it is most likely that unsaturated fatty acids are responsible for the formation of methyl ketones, unlike as suggested by the literature that methyl ketones originate from saturated fatty acids and 3-oxo esters [28,29]. In addition, with increasing heating time, the concentration of methyl ketones steadily increased (Figure 1). After 10 min, there was an especially strong increase of pentan-2-one, hexan-2-one and heptan-2-one. This is in line with the findings described by Schwartz et al. (1966), who showed that methyl ketones are progressively formed with increasing temperature and heating time in butter oil [26]. In the present study, decan-2-one could only be detected in very low amounts, originating at 10 min and therefore is not shown in Figure 1.

### 2.2. Formation of Methyl Ketones from Linoleic Acid

Most vegetable oils are rich in unsaturated linoleic acid, which is more likely oxidized and decomposed than saturated fatty acids or monounsaturated fatty acids. However, milk fat products additionally contain <1% proteins and there are phospholipids in both milk fat and vegetable oils [35,36,37]. Proteins and phospholipids like phosphatidylethanolamine have free amino groups that are highly reactive. In a model approach, the reactivity of linoleic acid without and with addition of 1,2-dipalmitoyl-*sn*-glycero-3-phosphoethanolamine (PE) at elevated temperature was investigated.

When heating 0.1% linoleic acid (mixed in an inert paraffin oil) at 160 °C without the addition of PE, mainly hexanal and only little amounts of the methyl ketones hexan-2-one and heptan-2-one were detected after 60 min. When adding PE, heptan-2-one was detected at 30 min with a strong increase during further heating. Hexan-2-one and hexanal could only be detected in small amounts (Table 2). Overall, about 10-times the amount of heptan-2-one was detected during the heating period of 90 min in the presence of PE. As less hexanal was detected at the same time, two possible conclusions can be drawn: (1) Hexanal plays a direct role in the formation of heptan-2-one, for example, by a methylation of hexanal or (2) The formation pathway of hexanal is inhibited by PE and the formation of heptan-2-one from the same precursor is promoted instead.

### 2.3. Degradation of Deca-2,4-dienal and the Influence of Amino Compounds

Already well-known from the literature, many VOCs are formed during lipid oxidation from linoleic acid. The two most common secondary lipid oxidation products of linoleic acid are deca-2,4-dienal, oct-2-enal and hexanal [38]. Thus, the degradation of these carbonyl compounds at an elevated temperature with and without amino compounds, such as PE was investigated to characterize their potential of forming heptan-2-one. As secondary degradation products are continuously formed during heat treatment and lipid oxidation, a degradation reaction to further compounds is difficult to detect. For this reason, *t*,*t*-deca-2,4-dienal was mixed in a paraffin oil as inert matrix and was treated under identical conditions as the oil samples.

**Figure 2:**
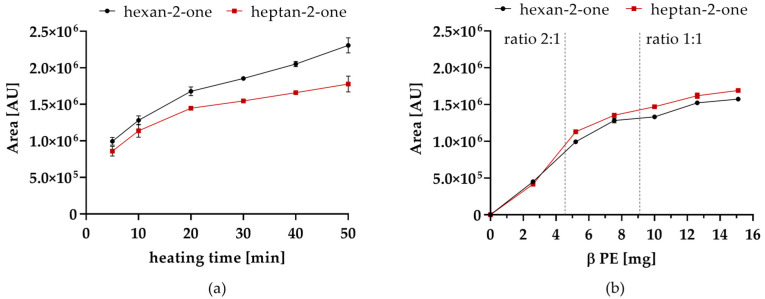
(**a**) Formation of hexan-2-one and heptan-2-one from 0.05% deca-2,4-dienal and PE (5 mg) at 160 °C; (**b**) Formation of hexan-2-one and heptan-2-one at 160 °C and 10 min treatment; 0.2% deca-2,4-dienal with varying levels of PE from a ratio of 3.5:1 up to 3:5.

Only hexanal was detected after 20 min, when heating deca-2,4-dienal without the addition of PE. At this sampling time, there was still 85% deca-2,4-dienal left. In the presence of PE, deca-2,4-dienal was completely degraded, as there was not any deca-2,4-dienal within 20 min of heat treatment left. Instead, the saturated aldehydes pentanal, hexanal and heptanal as well as the methyl ketones of hexan-2-one and heptan-2-one were found (Table 3). Although hexanal could be detected with and without PE addition, its concentration was considerably lower in the presence of PE. Furthermore, it was observed that the formation of methyl ketones over heating time (0–50 min) steadily increased (Figure 2a).

In addition, the influence of PE concentration on the reaction was investigated (Figure 2b). With increasing concentration of PE, higher amounts of methyl ketones were detected. Deca-2,4-dienal could not be detected after the equimolar ratio of deca-2,4-dienal and PE was exceeded. However, the increase of methyl ketones declines, when the molar ratio of deca-2,4-dienal and PE reached 2:1. Consequently, it is most likely that deca-2,4-dienal and PE react by forming an addition product resulting from two molecules deca-2,4-dienal and PE or decomposition products thereof.

Zhang and Ho (1989) investigated the degradation of deca-2,4-dienal with either cysteine or glutathione as amino compounds at 180 °C. In addition to the sulphur-containing VOC, they also observed the formation of hexan-2-one, heptan-2-one, octan-2-one and nonan-2-one in addition to the degradation of deca-2,4-dienal [39]. For this reason, the influence of different amino compounds on the degradation of *t*,*t*-deca-2,4-dienal was also investigated in the present study.

### 2.4. Influence of Different Amino Compounds

Based on the strong potential of PE to form new methyl ketones from deca-2,4-dienal, different kinds of amino compounds were chosen to investigate their reactivity based on structural characteristics. Due to the fact that PE also has emulsifying properties, 1,2-dipalmitoyl-*sn*-glycero-3-phosphocholine (PC) was used as emulsifier to avoid differing solubilities.

Table 4 shows the peak areas of hexan-2-one and heptan-2-one depending on the used amino compounds. After heat treatment for 20 min, no methyl ketones were detected in the experiments with l-glycine. l-Alanine and dl-leucine formed small amounts of heptan-2-one. Of the amino compounds used, only l-lysine and dl-glutamic acid were able to form both methyl ketones, whereas γ-aminobutyric acid (GABA) and ethanolamine formed the highest amounts of methyl ketones. However, slightly less hexan-2-one was detected with ethanolamine, but still twice as much heptan-2-one compared to PE.

The experiments showed that the amino acids that have only one free amino group at the alpha-position to the carboxyl group (here: l-glycine, l-alanine, and dl-leucine) form little to no methyl ketones, whereas the amino compounds with a terminal amino group (here: PE, ethanolamine, γ-amino butyric acid (GABA) and l-lysine) had the strongest reactivity, except for glutamic acid. As the latter can be decomposed to GABA at elevated temperatures by decarboxylation, methyl ketones in the sample with glutamic acid were most likely formed on a GABA basis. Additionally, the reactivity of PE is due to the structural characteristic of its ethanolamine group. Thus, it can be stated that amino acids can facilitate the formation of methyl ketones. However, this reaction is slow or only relevant to a lower extent. Rather, compounds with a terminal amino group that is not adjacent to a carboxyl group are able to form hexan-2-one and heptan-2-one from deca-2,4-dienal.

### 2.5. Formation of Methyl Ketones from Other Alka-2,4-dienals

**Figure 3:**
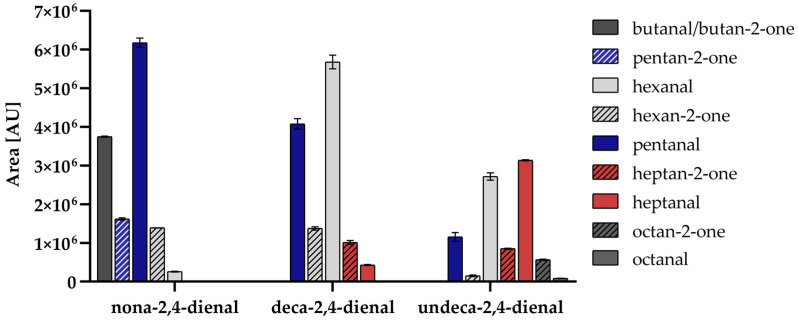
Peak areas of methyl ketones (stripes) and aldehydes (filled) from nona-2,4-dienal, deca-2,4-dienal and undeca-2,4-dienal (each 0.1% in paraffin oil) with PE, heated at 160 °C for 10 min and analyzed by HS-GC-MS.

To understand the systematics of the formation of methyl ketones, deca-2,4-dienal as well as nona-2,4-dienal and undeca-2,4-dienal were investigated in similar experiments with PE. Methyl ketones shortened by three and four C-atoms always resulted from the respective alka-2,4-dienals (nona-2,4-dienal → pentan-2-one and hexan-2-one; undeca-2,4-dienal → heptan-2-one and octan-2-one). In addition, small amounts of hexan-2-one (shortened by five C-atoms) were formed during heating of undeca-2,4-dienal, as well (Figure 3). Besides methyl ketones, the corresponding aldehydes also resulted from all alka-2,4-dienals. However, the methyl ketone shortened by three C-atoms was always formed in a higher extent than the corresponding aldehyde (for nona-2,4-dienal: hexan-2-one > hexanal). With the analytical separation method used, it was not possible to distinguish between butanal and butan-2-one, as these two substances have very similar retention times and electron ionization (EI) fragmentations.

### 2.6. The Reaction of Hexanal and 2-Butyloct-2-enal with Amino Compounds

Hexanal is a marker compound of lipid oxidation and can also be formed from deca-2,4-dienal. Consequently, hexanal was chosen to study the formation of methyl ketones from aldehydes. As GABA, PE, and ethanolamine showed the highest reactivity in the model systems with deca-2,4-dienal, GABA and PE were selected to investigate the influence of amino compounds on methyl ketone formation from hexanal, as well. In both systems, the methyl ketone hexan-2-one was detected and accompanied by a strong degradation of hexanal (Table 5). With PE, the aldol condensation product of hexanal, 2-butyloct-2-enal could be detected in higher amounts. Thus, PE promoted aldol condensation in a higher extent than GABA. 

After its initial formation within 5 min of heating time, the concentration of 2-butyloct-2-enal in both mixtures was decreasing, indicating subsequent reactions of this aldol product. This result is in line with the knowledge from the literature, describing that primary amine groups can catalyze the aldol condensation of aldehydes via intermediary Schiff bases [40]. As less 2-butyloct-2-enal but more hexan-2-one was detected in the GABA experiment, it can be assumed that the aldol condensation product and not hexanal itself is able to form hexan-2-one. To test this hypothesis, the reactivity of 2-butyloct-2-enal with amino compounds was also investigated. 2-Butyl-2-octenal reacted with both PE and GABA, forming hexan-2-one; peak areas were much larger than in the hexanal models. The GABA experiment showed a stronger degradation of 2-butyloct-2-enal but the formation of hexanal was observed mainly in the experiment with PE (Table 6).

Thus, both amino compounds were able to promote the formation of hexan-2-one from 2-butyloct-2-enal but PE also promoted other reactions that lead to the formation of hexanal. In summary, it was shown that the detection of methyl ketones from aldehydes seems to be caused by the formation of aldol condensation products from these aldehydes. 

### 2.7. The Reaction of Oct-2-enal with Amino Compounds

To investigate whether alk-2-enals are also capable of forming methyl ketones, oct-2-enal as a main degradation product of linoleic acid was analyzed. As PE, ethanolamine and GABA showed the highest reactivity with deca-2,4-dienal, these amino compounds were used in model experiments with oct-2-enal, as well.

Oct-2-enal reacted in the presence of PE to three different methyl ketones, namely hexan-2-one, heptan-2-one and octan-2-one, whereby lower quantities of methyl ketones could be detected than with deca-2,4-dienal. However, less octan-2-one was detected in the presence of ethanolamine and GABA, but the area of hexan-2-one was 8 (GABA) to 31 times (ethanolamine) higher than with PE (Table 7). Thus, under the influence of ethanolamine and GABA, hexan-2-one was formed predominantly. 

Due to different effects of the amino compounds on the formation of methyl ketones their formation depending on the molar ratio of oct-2-enal and PE was also investigated. An excess of oct-2-enal also caused a higher amount of methyl ketones in general but had no effect on the formation ratios of the methyl ketones. On the other hand, an increase in PE concentration had a particular effect on the amount of octan-2-one, which doubled with the molar ratio doubling as well from 1:1.8 to 1:3.6. 

As the formation of octan-2-one is comparatively low in the experiments with ethanolamine and GABA, it was assumed that different amino compounds catalyze the reaction mechanisms to methyl ketones to a different extent.

The experiments showed that a methyl ketone, which is two C-atoms shorter (hexan-2-one) and a methyl ketone, which is three C-atoms shorter (heptan-2-one), was formed from oct-2-enal and that the amino compounds had a different influence on the formation ratio of the methyl ketones. In any case, hexan-2-one was the preferred product. In addition, an increased formation of octan-2-one was observed with increasing PE concentration.

### 2.8. Systematic Studies on the Reaction of Alk-2-enals

To understand the systematics of the formation of methyl ketones from α,β-unsaturated aldehydes, a homologous series of alk-2-enals from hex-2-enal to undec-2-enal, as well as 2-butyloct-2-enal, was investigated. All alk-2-enals were able to form methyl ketones. In all experiments, methyl ketones of the same length (methyl ketones C_n_) or shorter by one C-atom (methyl ketones C_n−1_) or two C-atoms (methyl ketones C_n−2_) were detected, except for 2-undecanal, where no reaction to undecan-2-one was observed (Table 8).

For methodological reasons, it was not possible to distinguish between butanal and butan-2-one, as these two substances had very similar retention times and EI fragmentation patterns. However, a peak in the hex-2-enal experiment was detected that can be assigned to butanal or butan-2-one. From non-2-enal to undec-2-enale, the methyl ketones, which were shorter by three C-atoms, were detected in small amounts. The methyl ketone, which was shorter by four C-atoms, was detected in the undec-2-enal experiment. As mentioned before, the formation of the methyl ketone C_n_ of the same length increased with increasing PE concentration. This was especially valid for oct-2-enal and dec-2-enal, where methyl ketones C_n_ had the highest concentration. As the aldol condensation products, as well as the alk-2-enals have a double bond at position 2, they also reacted to methyl ketones. Compared to oct-2-enal, 2-butyloct-2-enal only reacted to one methyl ketone and hexan-2-one, most likely due to the branching at position 2.

**Figure 4:**
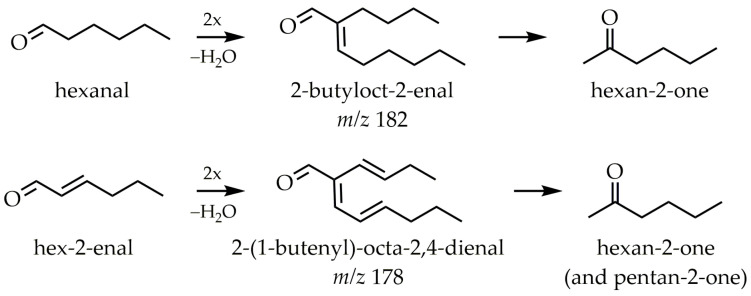
Reaction pathways of hexanal and hex-2-enal to the respective aldol condensation products and methyl ketones.

**Figure 5:**
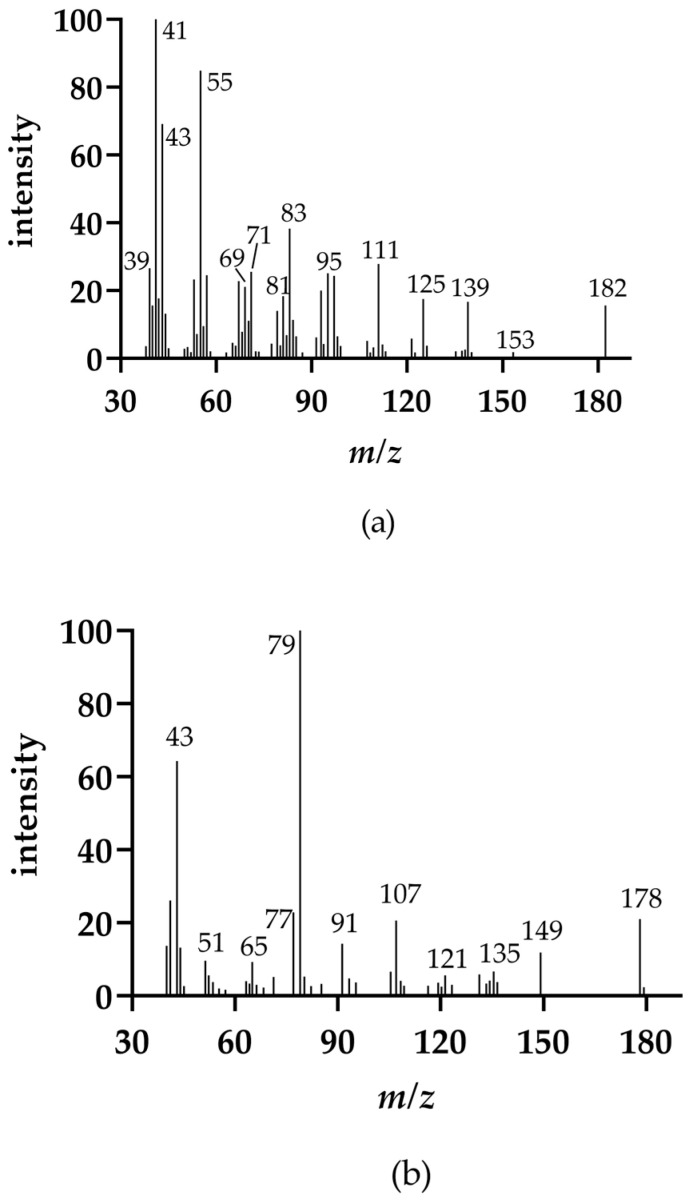
(**a**) EI spectrum of 2-butyloct-2-enal (commercially available standard compound); (**b**) EI spectrum of 2-(1-butenyl)-octa-2,4-dienal.

As shown before, PE catalyzed the aldol condensation and methyl ketone C_n_ (hexan-2-one) was formed from hexanal and the aldol condensation product 2-butyloct-2-enal, respectively. For this reason, it can be assumed that the methyl ketones C_n_ with the same number of C-atoms as the respective initial compounds are not formed directly from the initial compounds but from the respective aldol condensation products. This theory is strengthened, as in the experiment of hex-2-enal a reaction product with *m/z* 178, which can be assigned to the aldol condensation product, was detected (Figure 4). The EI fragmentation patterns of both aldol condensation products are shown in Figure 5. 2-Butyloct-2-enal was commercially available as a reference compound. The mass fragments with the highest intensities of both aldol condensation products differed by 4 Da each, due to the two additional double bonds of the 2-(1-butenyl)-octa-2,4-dienal. However, 2-(1-butenyl)-octa-2,4-dienal has not been mentioned as an aldol condensation product of hex-2-enal in the literature before.

For this reason, it is very likely that the above-mentioned increased formation of octan-2-one from oct-2-enal with increasing PE concentration was also due to the formation of the aldol condensation product from two molecules of oct-2-enal.

### 2.9. Methyl Ketone Formation Using the Compounds Deca-2,4-dienal, 2-decenal, and 4-decenal

As already mentioned, both double and monounsaturated compounds were able to form methyl ketones in different extents. To investigate whether the position of the double bond is essential for the formation of methyl ketones, the formation of methyl ketones from dec-2-enal, dec-4-enal, and deca-2,4-dienal as well as undec-8-enal was compared. In the experiments with undec-8-enal and PE at 160 °C, a degradation of undec-8-enal was observed, but no methyl ketones could be detected (data not shown).

It turned out that dec-4-enal, as well as deca-2,4-dienal, formed hexan-2-one and heptan-2-one, but additionally octan-2-one could be detected, which was also detected in the experiment with dec-2-enal (Table 9). Thus, the reaction of dec-4-enal is like the reaction of deca-2,4-dienal. This proves that, in the molecule deca-2,4-dienal, the more reactive functional group is mainly the second double bond at position 4 (as in dec-4-enal). In addition, the experiments prove that a double bond must be present in the initial molecule at position 2 or 4 to promote the reaction to methyl ketones.

## 3. Materials and Methods 

### 3.1. Chemicals and Materials

Hexanal, *trans*-hex-2-enal, *trans*-hept-2-enal, *trans*-oct-2-enal, *trans*-non-2-enal, *trans*-dec-2-enal, *trans*-undec-2-enal (≥95%), *trans*,*trans*-nona-2,4-dienal, *trans*,*trans*-deca-2,4-dienal, *trans*,*trans*-undeca-2,4-dienal (≥90%), *cis*-dec-4-enal, *cis*-undec-8-enal, hexan-2-one, heptan-2-one, nonan-2-one, 2-butyloct-2-enal, dl-alanine, l-glutamic acid, and ethanolamine were obtained from Merck KGaA, Darmstadt, Germany. Butan-2-one (>99%), pentan-2-one (>99%), octan-2-one (>98%), decan-2-one (>99%), 1,2-dipalmitoyl-sn-glycero-3-phosphoethanolamine (>96%), and 1,2-dipalmitoyl-sn-glycero-3-phosphocholine were obtained from TCI Chemicals Europe N.V., Zwijndrecht, Belgium. l-Glycine, l-lysine, dl-leucine, and γ-aminobutyric acid (97%) were obtained from Fisher Scientific GmbH, Schwerte, Germany. Paraffin oil (Pfeiffer^®^ P3) was obtained from MasCom Technologies GmbH, Bremen, Germany. Headspace vials (20 mL) and magnetic screw caps with a septum (SIL/PTFE 1.9 mm) were purchased from Chromatographie-Zubehör Trott, Kriftel, Germany. Fats and vegetable oils were purchased from local supermarkets. All chemicals were of analytical grade, if not mentioned otherwise.

### 3.2. Preparation of Model Systems and Experiments of Selected Fats and Oils

The respective compounds (aldehydes, alk-2-enals, and alka-2,4-dienals) were dissolved in inert paraffin oil with a final concentration of 0.05% with the exception of experiments with linoleic acid (0.1%), the experiments with nona-2,4-dienal, deca-2,4-dienal and undeca-2,4-dienal (each 0.1%), the experiments of deca-2,4-dienal (0.2%) with varying amounts of PE and the experiments of oct-2-enal (0.025%). One gram of the respective solution was weighed into a 20 mL-headspaces vial and mixed (Vortex-mixer VF2, IKA^®^-Werke GmbH & Co. KG, Staufen, Germany) for 15 s.

Optionally, 5 mg of amino compounds PE, PC, ethanolamine, γ-aminobutyric acid, dl-glutamic acid, l-lysine, dl-leucine, l-alanine, or l-glycine were added directly to the solution in the headspace vial. 10 milligrams of amino compounds were used in the experiments with hexanal, 2-butyloct-2-enal, dec-4-enal, and all alk-2-enals. 

The samples were incubated at 160 °C for 5–90 min (in the agitator module) and analyzed by headspace gas chromatography mass spectrometry (HS GC-MS). 

### 3.3. Analysis of Volatile Compounds with Static Headspace-GC-MS Analysis

For an automated sample incubation and application, the GC-MS system was equipped with a Combi-PAL-RSI autosampler from Axel Semrau GmbH & Co. KG, Sprockhövel, Germany. The agitator module incubated (160 °C) and agitated (250 rpm) the samples prior to GC injection. Subsequently, 1 mL of vapor space was injected into the GC−MS system consisting of a Shimadzu Deutschland GmbH (Duisburg, Germany) GC-17A gas chromatograph and a Shimadzu GCMS-QP5000 mass detector. The volatile compounds were separated using a Restek GmbH (Bad Homburg, Germany) Rtx^®^-Volatiles column (60 m × 0.25 mm, 1 μm film thickness). The following settings were used: carrier gas, helium; flow 1.00 mL/min; split 1:20; injection temperature 230 °C; interface temperature 230 °C; ion source temperature 200 °C; ionization energy 70 eV; temperature gradient 40 °C for 5 min, 10 °C/min to 150 °C, 2 °C/min to 210 °C. Under these conditions, it is possible to separate structural isomers like hexanal and hexan-2-one.

Chemical identification was confirmed by comparing retention times and mass spectra of samples with those of analytical standards and by using the NIST (National Institute of Standards and Technology) database except for 2-(1-butenyl)-octa-2,4-dienal, as mentioned in the discussion section. The quantitation was performed in SCAN mode (mass scan *m/z* 33–350) using total ion current (TIC) and expressed as abundance units (AU) and provide semiquantitative data. Data acquisition was performed using the GCMSsolution software version 1.20 (Shimadzu Deutschland GmbH, Duisburg, Germany).

### 3.4. Statistical Analysis

The samples were prepared and analyzed in triplicate. All results are shown as means ± standard deviation. All data were statistically analyzed using a one-way analysis of variance (ANOVA) to determine the effect of heating time or reactant on the formation of methyl ketones followed by a Dunnett or Tukey post-hoc test, respectively. The significance of the difference between two means was determined by the Student’s *t*-test. The analysis was performed using GraphPad Prism 8.0.2 software (San Diego, CA, USA).

## 4. Conclusions and Overview of Methyl Ketone Formation 

In conclusion, the experiments showed that methyl ketones can be formed from unsaturated fatty acids and their unsaturated secondary degradation products resulting from lipid oxidation. For this reason, methyl ketones can be considered as tertiary products of lipid oxidation. This pathway has not been described in the literature before and expands previous knowledge about the formation pathways from saturated fatty acids and 3-oxo acids.

**Figure 6:**
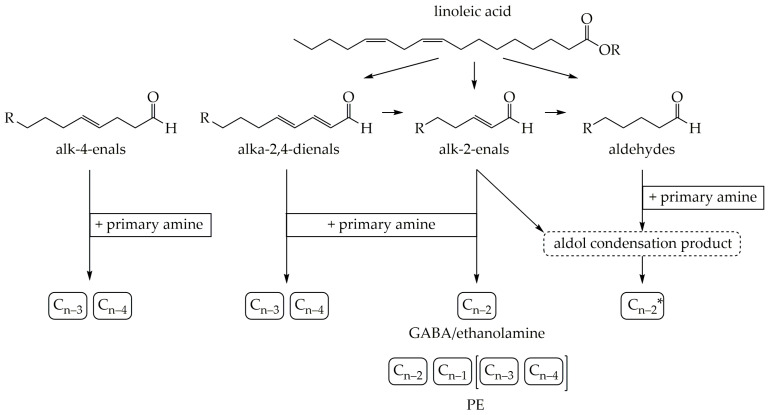
Systematic overview of the formation pathway of methyl ketones from linoleic acid and secondary degradation products; *: refers to the main alkyl chain length of the aldol condensation product; compounds in brackets have only been detected in some experiments.

The secondary degradation products of lipid oxidation can react in different ways to form methyl ketones (Figure 6). For instance, aldehydes and alk-2-enals react via the respective aldol condensation products to form the methyl ketones shortened by two C-atoms.

Furthermore, the position of the double bond in the reactant determines the chain length of the methyl ketone formed. A double bond at position 4 (alk-4-enals and alka-2,4-dienals) leads to methyl ketones shortened by three and four C-atoms. A double bond at position 2 (alk-2-enals and aldol condensation products) leads primarily to one and two C-atom shortened methyl ketones. Due to the branching in aldol condensation products, only the methyl ketone shortened by two C-atoms, is formed.

In addition, this study showed that primary amino groups promote the formation of methyl ketones to a different degree and influence the spectrum of formed products. For instance, ethanolamine showed the highest formation of methyl ketones and PE especially promotes the formation of octan-2-one, when heating oct-2-enal.

This study was able to prove that the main degradation products of linoleic acid, deca-2,4-dienal, oct-2-enal and hexanal, mainly produce the methyl ketones hexan-2-one, heptan-2-one and octan-2-one. This range of products was also found in fats and oils analyzed in this study, of which vegetable oils are rich in linoleic acids.

As mentioned above, methyl ketones are described in many food products. As almost all foods naturally contain lipids as well as amino compounds and are in many cases thermally treated during production and processing, the proposed pathways seem relevant for the formation of flavor-active methyl ketones in food.

## Figures and Tables

**Table 1:** Detected methyl ketones in milk fat and vegetable oils, heated at 160 °C 20 min; n. d. = not detected; substances in brackets were only detected with very low areas.

Sample	Methyl Ketones
butter	heptan-2-one, nonan-2-one, undecan-2-one
clarified butter	pentan-2-one, hexan-2-one, heptan-2-one, octan-2-one, nonan-2-one, decan-2-one, undecan-2-one
liquid vegetable fat preparation I (includes whey-based compounds)	heptan-2-one, octan-2-one, nonan-2-one, (undecan-2-one)
liquid vegetable fat preparation II	octan-2-one
sunflower oil virgin	hexan-2-one, heptan-2-one, octan-2-one
sunflower oil refined	hexan-2-one, heptan-2-one, octan-2-one
soy oil	(hexan-2-one), heptan-2-one, octan-2-one
rapeseed oil virgin	hexan-2-one, heptan-2-one, octan-2-one
rapeseed oil refined	heptan-2-one, octan-2-one
linseed oil	heptan-2-one, octan-2-one
hemp oil	(hexan-2-one), heptan-2-one, octan-2-one
corn oil	(heptan-2-one)
fat spread mix with butter	pentan-2-one, heptan-2-one, nonan-2-one, undecan-2-one
vegetable fat spread	heptan-2-one
fat spread (semi-fat)	n. d.
coconut fat	n. d.

**Table 2:** Comparison of peak areas of formed carbonyl compounds from 0.1% linoleic acid with 18:2 + (PE) and without PE (18:2) heated at 160 °C for 5–90 min and analyzed by HS-GC-MS; n. d. = not detectable.

Area [AU × 10^4^]	Heating Time	18:2	18:2 + PE
hexanal	5	81.83 ± 4.46 (a)	23.14 ± 13.01 (a)
	30	211.43 ± 4.66 (b)	27.10 ± 4.00 (a)
	60	271.94 ± 5.91 (c)	23.64 ± 3.47 (a)
	90	433.84 ± 46.81 (d)	14.41 ± 0.45 (a)
hexan-2-one	5	n. d.	n. d.
	30	n. d.	6.66 ± 0.75 (a)
	60	4.16 ± 1.11 (a)	9.21 ± 1.96 (b)
	90	9.38 ± 0.56 (b)	13.18 ± 0.99 (c)
heptan-2-one	5	n. d.	n. d.0 ± 0
	30	n. d.	104.73 ± 28.71 (a)
	60	8.00 ± 1.13 (a)	172.31 ± 15.45 (b)
	90	22.05 ± 5.09 (b)	227.25 ± 15.76 (c)

Statistically significant (*p* < 0.05) values within the columns are designated by different letters and refer in each case to the initial value of 5 min heating time (ANOVA; Dunnett’s test).

**Table 3:** Comparison of peak areas of formed carbonyl compounds from 0.05% *t,t*-deca-2,4-dienal each with and without PE after 20 min at 160 °C and analyzed by HS-GC-MS, n. d. = not detectable.

Area [AU × 10^5^]	*t,t*-Deca-2,4-dienal	*t,t*-Deca-2,4-dienal + PE
sum of aldehydes	95.10 ± 1.01	31.93 ± 1.18
pentanal	n. d.	12.41 ± 0.40
hexanal	95.10 ± 1.01	16.53 ± 0.74
heptanal	n. d.	2.98 ± 0.11
sum of methyl ketones	n. d.	31.26 ± 1.01
hexan-2-one	n. d.	16.79 ± 0.73
heptan-2-one	n. d.	14.47 ± 0.31
sum of isomers of deca-2,4-dienal	297.13 ± 14.07	n. d.
*c,t*-deca-2,4-dienal	10.16 ± 0.78	n. d.
*t,t*-deca-2,4-dienal	286.96 ± 14.46	n. d.

**Table 4:** Peak areas of hexan-2-one and heptan-2-one from deca-2,4-dienal (0.05% in paraffin oil) and different amino compounds, heated at 160 °C for 20 min and analyzed by HS-GC-MS, n. d. = not detected.

	Primary Amino Compound	Emulsifier	Area [AU × 10^5^]	
			Hexan-2-one	Heptan-2-one
PE (without *t,t*-deca-2,4-dienal)			n. d.	n. d.
*t,t*-deca-2,4-dienal +	PE	PE	16.79 ± 0.73 (a)	14.47 ± 0.31 (a)
	-	PC	n. d.	n. d.
	ethanolamine	PC	9.13 ± 0.76 (b)	38.99 ± 0.35 (b)
	γ-aminobutyric acid (GABA)	PC	25.76 ± 1.05 (c)	18.41 ± 1.99 (c)
	dl-glutamic acid	PC	7.67 ± 1.04 (b,d)	3.95 ± 1.08 (d)
	l-lysine	PC	6.22 ± 0.27 (d)	9.00 ± 0.33 (e)
	dl-leucine	PC	n. d.	3.27 ± 0.19 (d,f)
	l-alanine	PC	n. d	4.50 ± 0.47 (d,f,g)
	l-glycine	PC	n. d	n. d.

Statistically significant (*p* < 0.01) values within the columns of the same amino compound are designated by different letters (ANOVA; Tukey’s test).

**Table 5:** Peak areas of hexanal, hexan-2-one, and 2-butyloct-2-enal from hexanal (0.05% in paraffin oil) without or with γ-amino butyric acid (GABA) (10 mg) + PC or PE, heated at 160 °C for 40 min and analyzed by HS-GC-MS; n. d. = not detected.

Area [AU × 10^5^]	Heating Time	Hexanal	Hexan-2-one	2-Butyloct-2-enal
hexanal	5	5684.39 ± 461.65 (a)	n. d.	n. d.
	10	5809.61 ± 502.96 (a)	n. d.	n. d.
	20	6302.62 ± 841.65 (a)	n. d.	n. d.
	30	5835.05 ± 344.57 (a)	n. d.	n. d.
	40	5984.94 ± 246.96 (a)	n. d.	n. d.
hexanal + PC + GABA	5	1604.76 ± 120.64 (a)	10.35 ± 1.87 (a)	13.40 ± 9.75 (a)
	10	630.41 ± 36.81 (b)	25.90 ± 1.92 (b)	5.98 ± 1.46 (a)
	20	163.85 ± 45.47 (c)	38.77 ± 1.19 (c)	2.69 ± 0.98 (b)
	30	91.85 ± 6.68 (d)	43.08 ± 1.21 (d)	1.16 ± 0.22 (c)
	40	72.76 ± 8.14 (e)	48.00 ± 3.68 (e)	0.85 ± 0.11 (d)
hexanal + PE	5	432.36 ± 66.65 (a)	3.54 ± 0.15 (a)	69.26 ± 3.13 (a)
	10	154.74 ± 9.75 (b)	6.76 ± 0.77 (b)	49.12 ± 2.07 (b)
	20	95.25 ± 4.08 (c)	13.59 ± 0.16 (c)	28.22 ± 1.53 (c)
	30	72.26 ± 2.89 (d)	18.80 ± 1.30 (d)	17.40 ± 1.87 (d)
	40	56.37 ± 5.73 (e)	22.83 ± 0.20 (e)	12.30 ± 1.73 (e)

Statistically significant (*p* < 0.05) values within the columns are designated by different letters and refer in each case to the initial value of 5 min heating time (ANOVA; Dunnett’s test).

**Table 6:** Peak areas of 2-butyloct-2-enal, hexan-2-one, and hexanal from 2-butyloct-2-enal (0.05% in paraffin oil) without or with GABA + PC or PE, heated at 160 °C for 20 min and analyzed by HS-GC-MS; n. d. = not detected.

Area [AU × 10^5^]	2-Butyloct-2-enal	Hexan-2-one	Hexanal
2-butyloct-2-enal	686.34 ± 13.37 (a)	n. d.	16.07 ± 0.05 (a)
2-butyloct-2-enal + PC + GABA	384.56 ± 34.75 (b)	68.88 ± 6.94 (a)	n. d.
2-butyloct-2-enal + PE	80.66 ± 21.23 (c)	34.14 ± 3.93 (b)	173.26 ± 18.32 (b)

Statistically significant (*p* < 0.01) values within the columns of the same amino compound are designated by different letters (ANOVA; Tukey´s test).

**Table 7:** Peak areas of methyl ketones from oct-2-enal (0.05 and 0.025% in paraffin oil) with PE or ethanolamine and PC or GABA and PC, heated at 160 °C for 20 min and analyzed by HS-GC-MS; n. d. = not detected; n. a. = not analyzable, due to peak overlapping.

			Area [AU × 10^5^]		
	Amino Compound	Emulsifier	Hexan-2-one	Heptan-2-one	Octan-2-one
oct-2-enal	-	PC	n. d.	n. d.	n. d.
oct-2-enal 0.025%	PE 5 mg		2.76 ± 0.14 (a)	1.79 ± 0.21 (a)	2.87 ± 0.18 (a)
oct-2-enal 0.025%	PE 10 mg		3.55 ± 0.13 (a)	1.99 ± 0.06 (a)	4.81 ± 0.28 (b)
oct-2-enal 0.05%	PE 5 mg		5.28 ± 0.27 (b)	3.67 ± 0.09 (b)	4.84 ± 0.06 (b)
oct-2-enal 0.05%	PE 10 mg		6.76 ± 0.45 (c)	4.66 ± 0.30 (c)	10.10 ± 0.84 (c)
oct-2-enal 0.025%	ethanolamine	PC	113.75 ± 4.47 (a)	35.80 ± 5.80 (a)	2.47 ± 0.46 (a)
oct-2-enal 0.05%	ethanolamine	PC	216.04 ± 5.61 (b)	90.27 ± 14.49 (b)	6.38 ± 0.42 (b)
oct-2-enal 0.025%	GABA	PC	36.80 ± 0.44 (a)	n. a.	0.41 ± 0.11 (a)
oct-2-enal 0.05%	GABA	PC	53.87 ± 4.97 (b)	n. a.	0.68 ± 0.22 (a)

Statistically significant (*p* < 0.01) values within the columns of the same amino compound are designated by different letters (ANOVA; Tukey’s test); The significance of the difference between two means (ethanolamine, GABA) was determined by the Student’s *t*-test.

**Table 8:** Peak areas of methyl ketones from different alk-2-enals (0.05% in paraffin oil) with PE, heated at 160 °C for 20 min and analyzed by HS-GC-MS; n. d. = not detected.

Area [AU × 10^5^]	Hex-2-enal	Hept-2-enal	Oct-2-enal	Non-2-enal	Dec-2-enal	Undec-2-enal	2-Butyloct-2-enal
butan-2-one	n. d.	n. d.	n. d.	n. d.	n. d.	n. d.	n. d.
pentan-2-one	8.37 ± 0.55	10.06 ± 0.22	n. d.	n. d.	n. d.	n. d.	n. d.
hexan-2-one	3.18 ± 0.27	10.94 ± 0.66	5.22 ± 0.31	2.28 ± 0.18	n. d.	n. d.	33.24 ± 4.89
heptan-2-one	n. d.	1.52 ± 0.03	5.26 ± 0.50	3.53 ± 0.15	0.56 ± 0.02	1.75 ± 0.37	n. d.
octan-2-one	n. d.	n. d.	11.75 ± 1.59	3.31 ± 0.11	1.93 ± 0.31	2.19 ± 0.09	n. d.
nonan-2-one	n. d.	n. d.	n. d.	0.24 ± 0.17	0.93 ± 0.08	3.26 ± 0.22	n. d.
decan-2-one	n. d.	n. d.	n. d.	n. d.	3.40 ± 0.20	1.76 ± 0.17	n. d.

**Table 9:** Peak areas of methyl ketones from dec-2-enal, dec-4-enal, and deca-2,4-dienal (0.05% in paraffin oil) with PE, heated at 160 °C for 20 min and analyzed by HS-GC-MS; n. d. not detected.

Area [AU × 10^5^]	Dec-2-enal	Dec-4-enal	*t*,*t*-Deca-2,4-dienal
hexan-2-one	n. d.	9.77 ± 0.46	51.22 ± 0.52
heptan-2-one	1.96 ± 0.22	23.41 ± 1.55	32.39 ± 0.22
octan-2-one	4.88 ± 0.40	1.45 ± 0.10	n. d.
nonan-2-one	2.79 ± 0.26	n. d.	n. d.
decan-2-one	7.53 ± 1.35	n. d.	n. d.

## Data Availability

The data presented in this study are available on request from the corresponding author.

## Sample Availability

Samples of the compounds are available from the authors.

## References

[1] Kanzler C., Haase P.T. (2020). Melanoidins Formed by Heterocyclic Maillard Reaction Intermediates via Aldol Reaction and Michael Addition. J. Agric. Food Chem..

[2] van Boekel M., Fogliano V., Pellegrini N., Stanton C., Scholz G., Lalljie S., Somoza V., Knorr D., Jasti P.R., Eisenbrand G. (2010). A review on the beneficial aspects of food processing. Mol. Nutr. Food Res..

[3] Böttcher S., Steinhäuser U., Drusch S. (2015). Off-flavour masking of secondary lipid oxidation products by pea dextrin. Food Chem..

[4] Jacobsen C., Decker E.A. (2010). Understanding and reducing oxidative flavour deterioration in foods. Oxidation in Foods and Beverages and Antioxidant Applications: Woodhead Publishing Series in Food Science, Technology and Nutrition.

[5] Gómez-Cortés P., Sacks G.L., Brenna J.T. (2014). Quantitative analysis of volatiles in edible oils following accelerated oxidation using broad spectrum isotope standards. Food Chem..

[6] Dobarganes M.C., Márquez-Ruiz G., Erickson M.D. (2007). Formation and Analysis of Oxidized Monomeric, Dimeric, and Higher Oligomeric Triglycerides. Deep Frying (second edition).

[7] Frankel E.N., Selke E., Neff W.E., Miyashita K. (1992). Autoxidation of polyunsaturated triacylglycerols. IV. Volatile decomposition products from triacylglycerols containing linoleate and linolenate. Lipids.

[8] Widder S., Grosch W. (1997). Precursors of 2-nonenals causing the cardboard off-flavour in butter oil. Nahrung.

[9] Yasuhara A., Shibamoto T. (1995). Quantitative analysis of volatile aldehydes formed from various kinds of fish flesh during heat treatment. J. Agric. Food Chem..

[10] Yang J., Pan Z., Takeoka G., Mackey B., Bingol G., Brandl M.T., Garcin K., McHugh T.H., Wang H. (2013). Shelf-life of infrared dry-roasted almonds. Food Chem..

[11] Guillén M.D., Goicoechea E. (2008). Toxic oxygenated alpha,beta-unsaturated aldehydes and their study in foods: A review. Crit. Rev. Food Sci. Nutr..

[12] Perluigi M., Coccia R., Butterfield D.A. (2011). 4-Hydroxy-2-Nonenal, a Reactive Product of Lipid Peroxidation, and Neurodegenerative Diseases: A Toxic Combination Illuminated by Redox Proteomics Studies. Antioxid. Redox Signal..

[13] Frankel E.N., Frankel E.N. (2012). Chapter 4—Hydroperoxide decomposition. Lipid Oxidation: Oily Press Lipid Library Series.

[14] Chen G., Song H., Ma C. (2009). Aroma-active compounds of Beijing roast duck. Flavour Fragr. J..

[15] Coleman E.C., Ho C.-T., Chang S.S. (1981). Isolation and identification of volatile compounds from baked potatoes. J. Agric. Food Chem..

[16] Qian M., Nelson C., Bloomer S. (2002). Evaluation of fat-derived aroma compounds in blue cheese by dynamic headspace GC/Olfactometry-MS. J. Am. Oil Chem. Soc..

[17] Ghorbani Gorji S., Calingacion M., Smyth H.E., Fitzgerald M. (2019). Comprehensive profiling of lipid oxidation volatile compounds during storage of mayonnaise. J. Food Sci. Technol..

[18] Vara-Ubol S., Chambers E., Chambers D.H. (2004). Sensory characteristics of chemical compounds potentially associated with beany aroma in foods. J. Sens. Stud..

[19] Pionnier E., Engel E., Salles C., Le Quéré J.L. (2002). Interactions between non-volatile water-soluble molecules and aroma compounds in Camembert cheese. Food Chem..

[20] Carrapiso A.I., Ventanas J., García C. (2002). Characterization of the Most Odor-Active Compounds of Iberian Ham Headspace. J. Agric. Food Chem..

[21] Gu S.Q., Wu N., Wang X.C., Zhang J.J., Ji S.R. (2014). Analysis of Key Odor Compounds in Steamed Chinese Mitten Crab (Eriocheir sinensis). Adv. Mat. Res..

[22] Heiniö R.-L., Lehtinen P., Oksman-Caldentey K.-M., Poutanen K. (2002). Differences Between Sensory Profiles and Development of Rancidity During Long-Term Storage of Native and Processed Oat. Cereal Chem..

[23] Guillen M.D., Goicoechea E. (2008). Formation of oxygenated α,β-unsaturated aldehydes and other toxic compounds in sunflower oil oxidation at room temperature in closed receptacles. Food Chem..

[24] Macku C. (1991). Headspace volatile compounds formed from heated corn oil and corn oil with glycine. J. Agric. Food Chem..

[25] Smouse T.H., Chang S.S. (1967). A systematic characterization of the reversion flavor of soybean oil. J. Am. Oil Chem. Soc..

[26] Schwartz D.P., Parks O.W., Yoncoskie R.A. (1966). Quantitative studies on methyl ketone formation in butteroil: Effect of temperature. J. Am. Oil Chem. Soc..

[27] Langler J.E., Day E.A. (1964). Development and flavor properties of methyl ketones in milk fat. J. Dairy Sci..

[28] Crossley A., Heyes T.D., Hudson B.J.F. (1962). The effect of heat on pure triglycerides. J. Am. Oil Chem. Soc..

[29] Parks O.W., Keeney M., Katz I., Schwartz D.P. (1964). Isolation and characterization of the methyl ketone mecursor in butter fat. J. Lipid Res..

[30] Schwartz D.P., Spieglee P.S., Parks O.W. (1965). Effect of Water on Methyl Ketone Formation in Butteroil. J. Dairy Sci..

[31] van der Ven B., Begemann P.H., Schogt J.C.M. (1963). Precursors of methyl ketones in butter. J. Lipid Res..

[32] Markiewicz-Kęszycka M., Czyżak-Runowska G., Lipińska P., Wójtowski J. (2013). Fatty Acid Profile of Milk—A Review. Bull. Vet. Inst. Pulawy.

[33] Gardner H.W., Selke E. (1984). Volatiles from thermal decomposition of isomeric methyl (12S, 13S)-(E)-12,13-epoxy-9-hydroperoxy-10-octadecenoates. Lipids.

[34] Farmer L.J., Mottram D.S., Whitfield F.B. (1989). Volatile compounds produced in maillard reactions involving cysteine, ribose and phospholipid. J. Sci. Food Agric..

[35] Jensen R.G., Ferris A.M., Lammi-Keefe C.J. (1991). The Composition of Milk Fat1. J. Dairy Sci..

[36] U.S. Department of Agricuture (2019). FoodData Central. https://fdc.nal.usda.gov/.

[37] Aluyor E.O., Ozigagu C.E., Oboh O.I., Aluyor P. (2009). Chromatographic analysis of vegetable oils: A review. Sci. Res. Essays.

[38] Matthäus B., Decker E.A. (2010). 6-Oxidation of edible oils. Oxidation in Foods and Beverages and Antioxidant Applications: Woodhead Publishing Series in Food Science, Technology and Nutrition.

[39] Zhang Y., Ho C.T. (1989). Volatile compounds formed from thermal interaction of 2,4-decadienal with cysteine and glutathione. J. Agric. Food Chem..

[40] Pokorný J., Janitz W., Víden I., Velíšek J., Valentová H., Davídek J. (1987). Reaction of oxidized lipids with protein Part 14. Aldolization reactions of lower alkanals in presence of nonlipidic substances. Nahrung.

